# Selective deficiencies in descending inhibitory modulation in neuropathic rats: implications for enhancing noradrenergic tone

**DOI:** 10.1097/j.pain.0000000000001300

**Published:** 2018-06-29

**Authors:** Ryan Patel, Chaoling Qu, Jennifer Y. Xie, Frank Porreca, Anthony H. Dickenson

**Affiliations:** a Department of Neuroscience, Physiology & Pharmacology, University College London, London, United Kingdom; b Department of Pharmacology, University of Arizona, Tucson, AZ, United States

**Keywords:** In vivo electrophysiology, Ventral posterolateral thalamus, Conditioned place avoidance, Spinal nerve ligation, Neuropathic pain, Descending inhibition, Noradrenaline, Noradrenaline reuptake inhibitor, α2-adrenoceptor

## Abstract

Pontine noradrenergic neurones form part of a descending inhibitory system that influences spinal nociceptive processing. Weak or absent descending inhibition is a common feature of chronic pain patients. We examined the extent to which the descending noradrenergic system is tonically active, how control of spinal neuronal excitability is integrated into thalamic relays within sensory-discriminative projection pathways, and how this inhibitory control is altered after nerve injury. In vivo electrophysiology was performed in anaesthetised spinal nerve–ligated (SNL) and sham-operated rats to record from wide dynamic range neurones in the ventral posterolateral thalamus (VPL). In sham rats, spinal block of α_2_-adrenoceptors with atipamezole resulted in enhanced stimulus-evoked and spontaneous firing in the VPL, and produced conditioned place avoidance. However, in SNL rats, these conditioned avoidance behaviours were absent. Furthermore, inhibitory control of evoked neuronal responses was lost, but spinal atipamezole markedly increased spontaneous firing. Augmenting spinal noradrenergic tone in neuropathic rats with reboxetine, a selective noradrenergic reuptake inhibitor, modestly reinstated inhibitory control of evoked responses in the VPL but had no effect on spontaneous firing. By contrast, clonidine, an α_2_ agonist, inhibited both evoked and spontaneous firing, and exhibited increased potency in SNL rats compared with sham controls. These data suggest descending noradrenergic inhibitory pathways are tonically active in sham rats. Moreover, in neuropathic states, descending inhibitory control is diminished, but not completely absent, and distinguishes between spontaneous and evoked neuronal activity. These observations may have implications for how analgesics targeting the noradrenergic system provide relief.

## Abstract 2

Supplemental Digital Content is Available in the Text.

Descending noradrenergic pathways modulate spontaneous but not evoked thalamic neuronal hyperexcitability in neuropathic pain states. Spinal clonidine inhibits evoked and spontaneous firing, whereas reboxetine selectively inhibits evoked firing.

## 1. Introduction

Monoamine reuptake inhibitors are one of the commonly prescribed treatments for neuropathic pain, yet across different etiologies number needed to treat values only average around 6.4.^17^ Chronic pain conditions are frequently associated with a loss of endogenous inhibition,^68^ and clearly, enhancement of noradrenergic tone can be an effective treatment.^17,79^ Importantly, dividing patients into strata based on symptoms, which may reflect distinct underlying pathophysiological mechanisms, could improve patient outcomes.^13,60,79^

The importance of descending noradrenergic systems in pain chronicity is highlighted by the development of conditioned pain modulation (CPM) as a diagnostic tool as part of quantitative sensory testing. Conditioned pain modulation, a paradigm whereby heterotopic noxious stimulation reduces the pain percept of a test stimulus, provides a readout of the integrity of net endogenous inhibitory mechanisms. Patients with low CPM have increased propensity to develop chronic pain after surgery,^78^ and neuropathic patients with low CPM are more likely to respond to treatment with duloxetine (a serotonin and noradrenaline reuptake inhibitor [SNRI]) or tapentadol (a dual μ-opioid receptor agonist and noradrenaline reuptake inhibitor).^44,79^ These clinical observations have been successfully back-translated; diffuse noxious inhibitory controls, the analogous process to CPM in animals, are mediated by noradrenaline and are globally absent in neuropathic rats yet can be restored by tapentadol and reboxetine (an NRI),^3^ and the ability to engage descending inhibitory pathways influences severity of pain after nerve injury in rats.^12,75^ Inhibition of spinal α_2_-adrenoceptors also unmasks hypersensitivity in animals with no symptoms after nerve ligation and contralateral hypersensitivity.^12,28,75^ These studies suggest that noradrenergic pathways can act temporally and spatially to restrict spinal hypersensitivity and support a loss of inhibitory drive in neuropathic pain states.

Studies of descending modulation of pain typically use spinal endpoints, either reflexive or electrophysiological. The former assay is limited to examining withdrawal thresholds. The latter affords the ability to examine modulation of suprathreshold input, but the projection pathways of these neurones are rarely confirmed. Little is known about the impact of descending brainstem control of spinal excitability on neuronal activity in higher centres. To this end, we have studied the modulation of ascending spinal activity and subsequent integration into the ventral posterolateral thalamus (VPL), a key sensory discriminative relay. During neurosurgical procedures, microstimulation of the ventralis caudalis can evoke thermal and mechanical sensations.^37,46^ Furthermore, polymodal neurones within this lateral thalamic pathway have the capacity to encode stimulus intensity and have been characterised across species, including humans.^1,33,80^ Unlike spinal neuronal responses after peripheral nerve injury in rats,^9,48,51^ VPL wide dynamic range (WDR) neurones exhibit higher rates of spontaneous firing and exaggerated responses to evoked stimulation.^21,50,69^ These electrophysiological recordings allow for a linear correlation of stimulus intensity and response, and can provide a sensory neuronal correlate of evoked hypersensitivity after injury.^45^ In an attempt to define drug effects on affective state, we additionally perform conditioned place avoidance (CPA) testing as this assay has the advantage of providing insight into the rewarding or aversive nature of pharmacological interventions.

## 2. Methods

### 2.1. Animals

Naïve, sham-operated, and spinal nerve–ligated (SNL) (14-20 days after surgery) male Sprague-Dawley rats (250-350 g) were used for behavioural and electrophysiological experiments (Harlan, Indianapolis, IN and Biological Services, University College London, United Kingdom, respectively). Animals were group-housed (maximum of 4) on a conventional 12 hours:12 hours light-dark cycle; food and water were available ad libitum. Temperature and humidity of holding rooms were closely regulated. Electrophysiological and surgical procedures described here were approved by the UK Home Office and adhered to the Animals (Scientific Procedures) Act 1986. Behavioural and surgical procedures were approved by the Institutional Animal Care and Use Committee of the University of Arizona. All experiments were designed in accordance with International Association for the Study of Pain ethics guidelines.^82^

### 2.2. Spinal nerve ligation surgery

Spinal nerve ligation surgery was performed as previously described.^26^ Rats (130-140 g) were maintained under 2% vol/vol isoflurane anaesthesia delivered in a 3:2 ratio of nitrous oxide and oxygen. Under aseptic conditions, a paraspinal incision was made and the tail muscle excised. Part of the L5 transverse process was removed to expose the left L5 and L6 spinal nerves, which were then isolated with a glass nerve hook (Ski-Ry, London, United Kingdom), and ligated with a nonabsorbable 6-0 braided silk thread proximal to the formation of the sciatic nerve. The surrounding skin and muscle was closed with absorbable 4-0 sutures. Sham surgery was performed in an identical manner omitting the nerve hook/ligation step. All rats groomed normally and gained weight in the following days after surgery.

### 2.3. Intrathecal cannulation

For behavioural studies, intrathecal catheters were implanted 5 to 7 days before sham or spinal nerve ligation surgery as described before.^73,77^ Rats were anaesthetised with intraperitoneal ketamine (80 mg/kg)/xylazine (12 mg/kg). After aseptic preparation, the atlanto-occipital membrane was exposed and incised. A 7.5-cm long PE-10 catheter was slowly inserted into the intrathecal space through the membrane to the lumbar spinal cord. The catheter was secured inside the muscle, and the skin was closed with wound clips. The animals were single-housed after recovery. Animals exhibiting signs of paralysis (<5%) were removed from the study.

### 2.4. Paw withdrawal thresholds

Baseline thresholds were determined 1 day before sham or spinal nerve ligation surgery. Rats were placed in isolation inside chambers on a wire mesh floor and allowed to acclimatise. The withdrawal threshold of the hind paw was measured in response to probing of the plantar surface with a series of calibrated von Frey filaments (Stoelting, Wood Dale, IL) in logarithmically spaced increments ranging from 0.41 to 15 g. Each filament was applied perpendicularly to the plantar surface of the left hind paw for 3 seconds; flinching, licking, shaking, or other directed behaviours were considered positive responses. Withdrawal threshold was determined by sequentially increasing or decreasing the stimulus force (starting with the 2-g filament) using the “up-down” method as previously described.^8^ Pharmacology was performed on postoperative day 17; withdrawal thresholds were determined before dosing and subsequently at 20, 40, and 60 minutes after dosing. The experimenter was blinded to treatment group; rats were randomised into groups (by an independent person), and received either intrathecal vehicle (5-μL normal saline) or atipamezole (20 μg/5 μL).

### 2.5. Conditioned place avoidance

A single-trial CPA protocol was performed as previously described.^32^ On preconditioning day (day 13 after spinal nerve ligation/sham surgery), rats were placed into the CPA boxes with access to all chambers, and time spent in each chamber over 15 minutes was determined by an automated process. To assure no chamber preference bias before conditioning, animals spending more than 80% (720 seconds) or less than 20% (180 seconds) of the total time in a chamber were eliminated from further testing. Chamber pairings were counterbalanced between the control and drug chambers. The following day, all rats received 5-μL vehicle (normal saline) intrathecally and were immediately placed into one randomly assigned conditioning chamber for 30 minutes without access to the other chamber. Four hours later in the afternoon, rats received intrathecal atipamezole (20 μg/5 μL) paired with the opposite chamber for 30 minutes. On test day (day 15 after spinal nerve ligation/sham surgery), 20 hours after the afternoon pairing, rats were placed in the apparatus with access to all chambers again, and their behaviour was recorded for 15 minutes for automated analysis of chamber preference/avoidance. The difference score was calculated by subtracting the baseline time from the test time. The order of pairing with morning saline and afternoon atipamezole avoided possible effects of extended duration of action of the drug with learning and is consistent with our previous methods.^32^

### 2.6. In vivo electrophysiology

**Figure 1.**
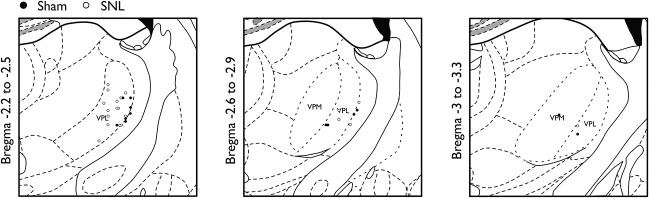
Recording sites within the ventral posterolateral thalamus from 13 sham and 20 neuropathic rats. Filled circles (●) represent sham, and open circles (○) represent spinal nerve–ligated (SNL) rat experiments.

Thalamic neuronal recordings were performed as previously described.^50^ The experimenter was not blind to the injury or drug administration. Animals were initially anaesthetised with 3.5% vol/vol isoflurane delivered in 3:2 ratio of nitrous oxide and oxygen. Once areflexic, a tracheotomy was performed and rats were subsequently maintained on 1.5% vol/vol isoflurane for the remainder of the experiment. Rats were secured in a stereotaxic frame, and after exposure of the skull, coordinates for the right ventral posterolateral (VPL) thalamus (contralateral to injury) were calculated in relation to bregma (2.28-mm caudal and 3.2-mm lateral).^70^ A small craniotomy was performed with a high-speed surgical microdrill. The muscle overlying the lumbar vertebrae was removed, a partial laminectomy was performed to expose the L4 to L6 lumbar region, and the overlying dura was removed. Once haemostasis was achieved, the surrounding muscle was coated in petroleum jelly to form a hydrophobic barrier to contain the drug. Extracellular recordings were made from VPL thalamic neurones with receptive fields on the glabrous skin of the left paw hind toes (see Fig. 1 for stereotaxically determined recording sites) using 127-µm diameter 2-MΩ parylene-coated tungsten electrodes (A-M Systems, Sequim, WA). The receptive field was stimulated using a range of natural stimuli (brush, von Frey filaments—2, 8, 15, 26, and 60 g and heat—35, 42, 45, and 48°C) applied over a period of 10 seconds per stimulus. The heat stimulus was applied with a constant water jet onto the centre of the receptive field. Acetone and ethyl chloride (100 μL) were applied as an evaporative innocuous cooling and noxious cooling stimulus, respectively,^34^ and responses quantified over 10 seconds after application. Evoked responses to room temperature water (25°C) were minimal, or frequently completely absent, and subtracted from acetone-evoked and ethyl chloride–evoked responses to control for any concomitant mechanical stimulation during application. Stimuli were applied starting with the lowest intensity stimulus with approximately 40 seconds between stimuli in the following order: brush, von Frey, cold, and heat.

Animals were under anaesthesia for 4 to 6 hours. This included obtaining baseline responses over the course of an hour and determining drug effects over 2 hours. Baseline recordings were stable over the first hour of recording; however, subsequent time-dependent or order-dependent effects of drug dosing cannot be ruled out over the remainder of the experiment. Baseline recordings were made with 25-µL vehicle applied topically to the dorsal aspect of the spinal cord (after aspiration of any cerebrospinal fluid), hence all observed effects can be attributed to drug actions. After 3 baseline trials (stimuli applied in the order described above with 5 minutes between each set of trials, data were averaged to give control values), the vehicle was removed, and 50-µg and 100-µg atipamezole (Sigma, Gillingham, United Kingdom), or 10-µg and 50-µg reboxetine mesylate (Tocris, Abingdon, United Kingdom) were cumulatively applied to the spinal cord in a volume of 25 µL (vehicle: 97% normal saline, 2% cremophor, 1% dimethylsulfoxide), and neuronal responses were characterised 20 and 40 minutes after dosing. For clonidine studies, 50- and 100-µg clonidine hydrochloride (Sigma) were applied to the cord in a volume of 25 µL (vehicle: normal saline), and neuronal responses were characterised 10 and 30 minutes after dosing; time point of peak change from baseline is plotted. The second dose was applied approximately 50 to 60 minutes after aspiration of the first dose; excess drug was washed from the cord with 25-µL vehicle (applied for 2-3 minutes). Drug doses were guided by previous studies,^3,7,27,54,64^ and their effects were tested in a pilot study.

Data were captured and analysed by a CED1401 interface coupled to a computer with Spike2 v6 software (Cambridge Electronic Design, Cambridge, United Kingdom) with rate functions. The signal was amplified (×6000), bandpass filtered (low-/high-frequency cutoff 1.5/2 kHz), and digitised at rate of 20 kHz. Spike sorting was performed post hoc with Spike2 using fast Fourier transform followed by 3-dimensional principal component analysis of waveform feature measurements for multiunit discrimination. Neurones were recorded from one site per rat; 1 to 3 neurones were characterised at each site. Stimulus-evoked neuronal responses were determined by subtracting total spontaneous neuronal activity in the 10-second period immediately preceding stimulation. Spontaneous firing of individual neurones (number of spikes per second) is expressed as the mean of these 10-second periods. Burst firing (number of bursts per second) was determined over a period of 60 seconds in the absence of stimulation as previously described.^23^ Burst parameters were maximum initial interval signifying burst onset (6 ms), longest interspike interval allowed within burst (9 ms), and minimum number of events in a burst (2).

### 2.7. Statistics

Statistical analyses were performed using SPSS v25 (IBM, Armonk, NY). Heat and mechanical coding of neurones were compared with a 2-way repeated-measures (RM) analysis of variance (ANOVA), followed by a Bonferroni post hoc test for paired comparisons. Cold, brush, and spontaneous/burst firing were compared with a 1-way RM ANOVA, followed by a Bonferroni post hoc test for paired comparisons. Conditioned place avoidance measures were compared with a 2-way RM ANOVA, followed by a Bonferroni post hoc test for paired comparisons, whereas difference scores were compared with an unpaired Student *t* test. Where appropriate, sphericity was tested using Mauchly test; the Greenhouse-Geisser correction was applied if violated. Paw withdrawal time courses were compared with the Friedman test, followed by a Wilcoxon post hoc and Bonferroni correction for paired comparisons. Minimum group sizes were determined by a priori calculations (α 0.05, 1 − β 0.8). All data represent mean ± 95% confidence interval. *P < 0.05, **P < 0.01, ***P < 0.001.

## 3. Results

### 3.1. Spinal α_2_-adrenoceptor inhibition has no effect on stimulus-evoked withdrawal responses in sham and spinal nerve–ligated rats

**Figure 2.**
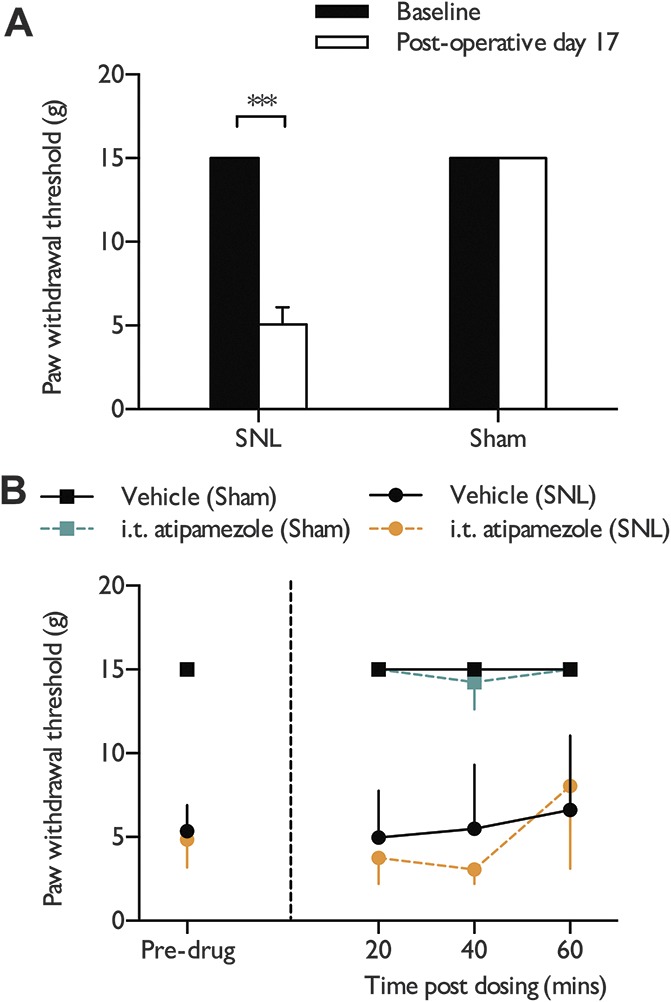
Spinal α_2_-adrenoceptor inhibition does not alter withdrawal thresholds in sham and SNL rats. Mechanically evoked withdrawal thresholds in sham (n = 29) and SNL rats (n = 14) before surgery and on postoperative day 17 (A). Paw withdrawal thresholds before and after intrathecal delivery of either vehicle (sham: n = 13, SNL n = 6) or atipamezole (sham: n = 16, SNL n = 8) (B). Data represent mean ± 95% CI. ***P < 0.001. CI, confidence interval; i.t., intrathecal; SNL, spinal nerve–ligated.

To determine the extent of any descending inhibitory control of hind paw withdrawal responses, the α_2_-adrenoceptor antagonist atipamezole (20 μg/5 μL) was delivered intrathecally to sham and SNL rats, and withdrawal thresholds to punctate mechanical stimulation quantified. In sham-operated rats, withdrawal thresholds after surgery (day 17) were comparable with baseline responses (Wilcoxon test, *P* = 1.00), whereas SNL rats exhibited mechanical hypersensitivity after peripheral nerve injury (Wilcoxon test, *P* = 0.00098) (Fig. 2A). Neither intrathecal vehicle nor atipamezole altered withdrawal thresholds in sham-operated rats (Friedman test, vehicle: *P* = 1.00, atipamezole: *P* = 0.392). Similarly, in SNL rats neither vehicle nor atipamezole had any overall effect on established mechanical hypersensitivity (Friedman test, vehicle: *P* = 0.478, atipamezole: *P* = 0.069) (Fig. 2B).

### 3.2. Wide dynamic range neurones in the VPL exhibit hyperexcitability in spinal nerve–ligated rats

Behavioural observations were extended to include a wider range of modalities and intensities by characterising sensory neuronal processing in the VPL. Wide dynamic range neurones were identified on the basis of responses to brush, noxious punctate mechanical stimulation, and noxious thermal stimulation of the receptive field. After obtaining stable baseline responses, pharmacological studies were performed. A total of 49 neurones were characterised; Table 1 summarises predrug baseline responses to evoked stimulation. Wide dynamic range neurones in SNL rats exhibited greater stimulus-evoked responses, and higher rates of spontaneous and burst firing consistent with our previous observations.^50^ In a separate study, this elevated spontaneous firing rate was inhibited by spinal and intraplantar lidocaine in SNL rats (data not shown) implying ongoing activity in sensory projection pathways, although we cannot conclude whether this represents noxious activity.

**Table 1:**
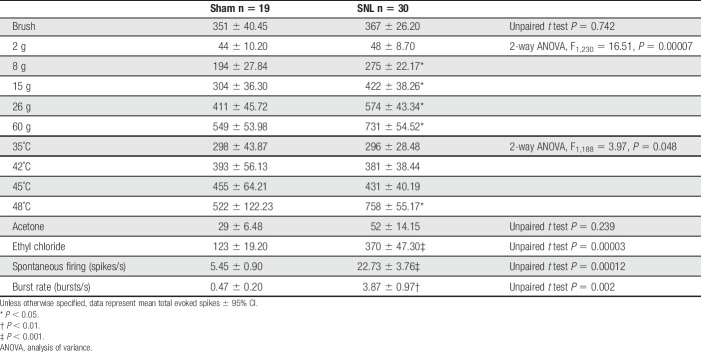
Baseline characterisations of wide dynamic range neurones in the ventral posterolateral thalamus of naïve/sham and spinal nerve–ligated (SNL) rats.

### 3.3. Spinal α_2_-adrenoceptor inhibition reveals tonic noradrenergic inhibitory tone in naïve/sham rats

**Figure 3.**
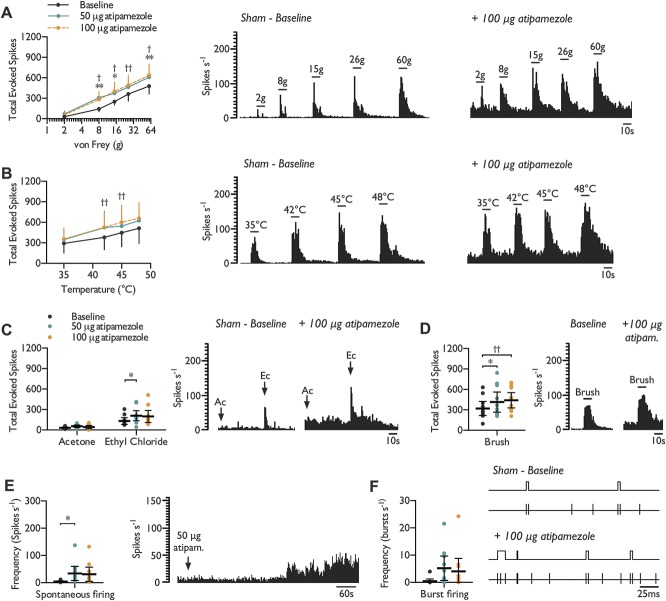
Inhibiting spinal α_2_-adrenoceptors increases stimulus-evoked and spontaneous firing in the VPL of naïve/sham rats. Wide dynamic range (WDR) neuronal responses to punctate mechanical (A), heat (B), cold (C), dynamic brush (D) stimuli, and spontaneous (E) and burst firing rates (F), before and after spinal administration of atipamezole. Histogram and event mark traces represent typical single-unit responses. Data represent mean ± 95% CI, n = 11 neurones from 8 rats. *Difference between baseline and 50-μg atipamezole, †difference between baseline and 100-μg atipamezole, *P < 0.05, **P < 0.01. Ac, acetone; CI, confidence interval; Ec, ethyl chloride.

After application of 50- and 100-μg atipamezole to the spinal cord, inhibition of α_2_-adrenoceptors increased evoked thalamic neuronal responses to low intensity (8 g) and to intensities that may reach and then exceed noxious levels (15, 26, and 60 g) of punctate mechanical stimulation (2-way RM ANOVA *P* = 0.000096, F_2,20_ = 15.21) (Fig. 3A). In addition, heat-evoked responses were increased at noxious intensities of stimulation (2-way RM ANOVA *P* = 0.019, F_2,20_ = 4.899) (Fig. 3B). Acetone and ethyl chloride were applied as innocuous and noxious evaporative cooling stimuli, respectively. Neuronal responses to innocuous cooling were unaffected by spinal atipamezole (1-way RM ANOVA *P* = 0.144, F_1.28,12.79_ = 2.383); however, noxious cold-evoked responses were facilitated (1-way RM ANOVA *P* = 0.016, F_2,20_ = 5.096) (Fig. 3C). Dynamic brush-evoked responses were also enhanced after dosing (1-way RM ANOVA *P* = 0.007, F_2,20_ = 6.49) (Fig. 3D). Spontaneous activity increased in 8/11 units within 5 to 10 minutes of drug application (1-way RM ANOVA *P* = 0.028, F_1.13,11.30_ = 6.079) (Fig. 3E), and this increased activity was sustained for the duration of the experiment. However, there was no overall effect on the burst rate (1-way RM ANOVA *P* = 0.069, F_1.14,11.37_ = 3.926) (Fig. 3F). Effect sizes in naïve (n = 4 neurones from 3 rats) and sham animals (n = 7 neurones 5 rats) were comparable and thus were pooled for analysis.

### 3.4. Spinal α_2_-adrenoceptor inhibition reveals a selective loss of descending inhibitory control of evoked neuronal responses in spinal nerve–ligated rats

**Figure 4.**
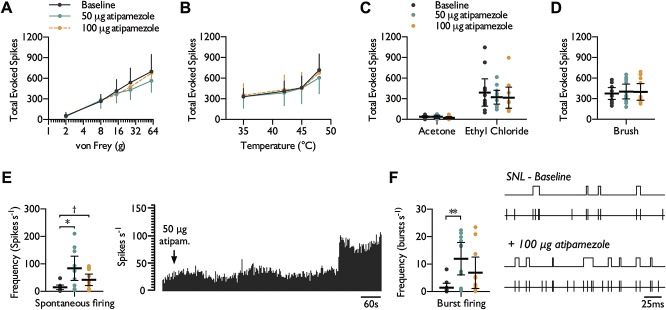
Inhibiting spinal α_2_-adrenoceptors selectively increases spontaneous firing in the VPL of SNL rats. Wide dynamic range (WDR) neuronal responses to punctate mechanical (A), heat (B), cold (C), dynamic brush (D) stimuli, and spontaneous (E) and burst firing rate (F), before and after spinal administration of atipamezole. Histogram and event mark traces represent typical single-unit responses. Data represent mean ± 95% CI, n = 11 neurones from 7 rats. *Difference between baseline and 50-μg atipamezole, †difference between baseline and 100-μg atipamezole, *P < 0.05, **P < 0.01. CI, confidence interval; SNL, spinal nerve–ligated.

Unlike in sham-operated rats, 50- and 100-μg atipamezole had no effect on punctate mechanical (2-way RM ANOVA *P* = 0.432, F_2,20_ = 0.876) (Fig. 4A), heat (2-way RM ANOVA *P* = 0.848, F_2,20_ = 0.167) (Fig. 4B), cooling (acetone: 1-way RM ANOVA *P* = 0.226, F_2,20_ = 1.604; ethyl chloride: 1-way RM ANOVA *P* = 0.370, F_2,20_ = 1.047) (Fig. 4C), and brush-evoked neuronal responses in the VPL of SNL rats (1-way RM ANOVA *P* = 0.858, F_2,20_ = 0.154) (Fig. 4D). Surprisingly, in contrast to the lack of effect on evoked responses, spontaneous activity (1-way RM ANOVA *P* = 0.0039, F_1.04,10.39_ = 13.415) (Fig. 4E) and burst firing (1-way RM ANOVA *P* = 0.00079, F_2,20_ = 10.428) (Fig. 4F) substantially increased after spinal delivery of atipamezole.

### 3.5. Spinal α_2_-adrenoceptor inhibition induces conditioned place avoidance in sham but not spinal nerve–ligated rats

**Figure 5.**
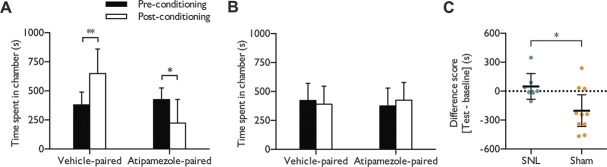
Inhibiting spinal α_2_-adrenoceptors produces avoidance learning behaviour in sham but not SNL rats. Time spent in respective drug-paired chambers before and after conditioning with vehicle and atipamezole in sham rats (n = 10) (A). Time spent in respective drug-paired chambers before and after conditioning with vehicle and atipamezole in SNL rats (n = 7) (B). Difference score (atipamezole-paired chamber) for SNL and sham rats (C). Data represent mean ± 95% CI. *P < 0.05, **P < 0.01. CI, confidence interval; SNL, spinal nerve–ligated.

In light of the aforementioned observations, we next examined whether inhibiting spinal α_2_-adrenoceptors produced avoidance learning behaviours in sham and SNL rats. The time spent in atipamezole (20 μg/5 μL) and vehicle-paired chambers during preconditioning trials were comparable across groups. After conditioning, most sham-operated animals (7/10) displayed clear avoidance of the atipamezole-paired chamber, and a corresponding increase in the time spent in the vehicle-paired chamber was observed (2-way RM ANOVA *P* = 0.0033, F_1,9_ = 15.599) (Fig. 5A). By contrast, SNL rats displayed no preference for the vehicle- or atipamezole-paired chamber after a single conditioning trial (2-way RM ANOVA *P* = 0.502, F_1,6_ = 0.511) (Fig. 5B) or after 2 conditioning trials (data not shown). The difference scores (atipamezole-paired chamber) further illustrate the larger effect size in sham rats compared with SNL (unpaired Student's *t* test, *t* = 2.54, *df* = 15, *P* = 0.0227) (Fig. 5C).

### 3.6. Activation of spinal α_2_-adrenoceptors attenuates evoked and spontaneous firing in the VPL in sham and spinal nerve–ligated rats

**Figure 6.**
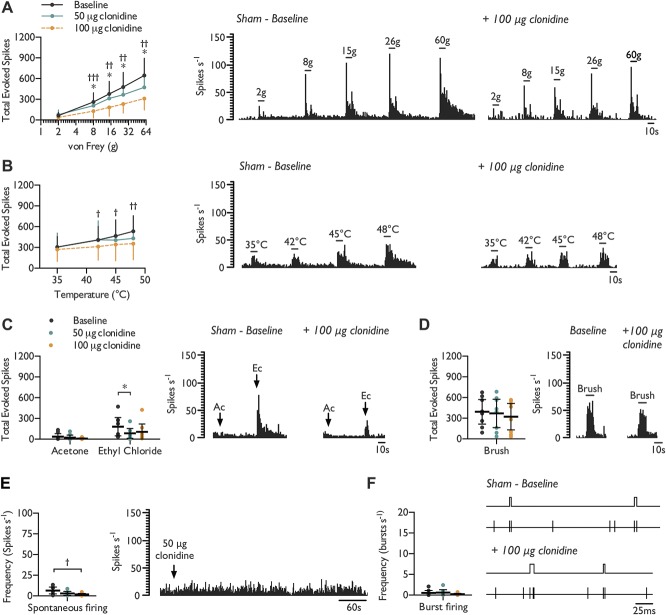
Activating spinal α_2_-adrenoceptors reduces stimulus-evoked and spontaneous firing in the VPL of sham rats. Wide dynamic range (WDR) neuronal responses to punctate mechanical (A), heat (B), cold (C), dynamic brush (D) stimuli, and spontaneous (E) and burst firing rate (F), before and after spinal administration of clonidine. Histogram and event mark traces represent typical single-unit responses. Data represent mean ± 95% CI, n = 8 neurones from 5 rats. *Difference between baseline and 50-μg clonidine, †difference between baseline and 100-μg clonidine, *P < 0.05, **P < 0.01, ***P < 0.001. Ac, acetone; Ec, ethyl chloride; CI, confidence interval.

Spinal clonidine (50 and 100 μg), an α_2_-adrenoceptor agonist, dose dependently inhibited evoked and spontaneous thalamic neuronal firing. In sham rats, responses to punctate mechanical (2-way RM ANOVA *P* = 0.000005, F_2,14_ = 32.636) (Fig. 6A) and heat stimuli (2-way RM ANOVA *P* = 0.00265, F_2,14_ = 9.342) (Fig. 6B) were reduced across intensities of stimulation. Responses to innocuous cooling were unaffected, although noxious cooling-evoked responses were reduced by clonidine (acetone: 1-way RM ANOVA *P* = 0.163, F_1.07,7.49_ = 2.399; ethyl chloride: 1-way RM ANOVA *P* = 0.0109, F_2,14_ = 6.356) (Fig. 6C). We found weak evidence for an overall decrease in brush-evoked firing (1-way RM ANOVA *P* = 0.0411, F_2,14_ = 4.044, paired comparisons *P* > 0.05) (Fig. 6D). Low rates of spontaneous firing were observed and were only inhibited at the highest dose tested (1-way RM ANOVA *P* = 0.0171, F_1.21,8.49_ = 8.12) (Fig. 6E). Likewise, the levels of burst firing were minimal but were not altered by clonidine (1-way RM ANOVA *P* = 0.115, F_2,14_ = 2.529) (Fig. 6F).

**Figure 7.**
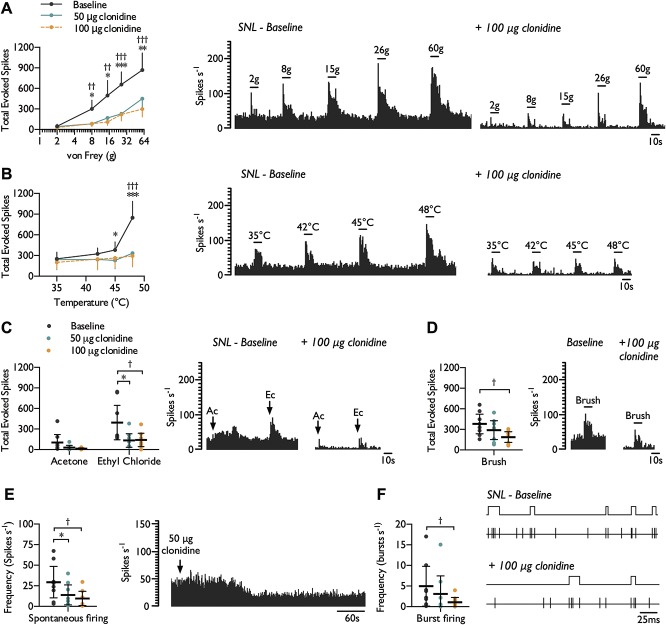
Activating spinal α_2_-adrenoceptors reduces stimulus-evoked and spontaneous firing in the VPL of SNL rats. Wide dynamic range (WDR) neuronal responses to punctate mechanical (A), heat (B), cold (C), dynamic brush (D) stimuli, and spontaneous (E) and burst firing rate (F), before and after spinal administration of clonidine. Histogram and event mark traces represent typical single-unit responses. Data represent mean ± 95% CI, n = 8 neurones from 6 rats. *Difference between baseline and 50-μg clonidine, †difference between baseline and 100-μg clonidine, *P < 0.05, **P < 0.01, ***P < 0.001. Ac, acetone; Ec, ethyl chloride; CI, confidence interval; SNL, spinal nerve–ligated.

In SNL rats, spinal clonidine exhibited enhanced inhibitory effects at both doses tested, particularly on mechanical and heat-evoked responses (Figs. 7A–F and Supplementary Table 1; available online at http://links.lww.com/PAIN/A596). Neuronal responses to punctate mechanical (2-way RM ANOVA *P* = 0.000003, F_2,14_ = 36.573) (Fig. 7A) and heat stimuli (2-way RM ANOVA *P* = 0.000046, F_2,14_ = 22.11) (Fig. 7B) were substantially decreased compared with baseline, again across intensities of stimulation. There was no overall effect on responses to innocuous cooling; however, noxious cold evoked markedly fewer spikes (acetone: 1-way RM ANOVA *P* = 0.092, F_2,14_ = 2.844; ethyl chloride: 1-way RM ANOVA *P* = 0.00786, F_1.05,7.37_ = 12.79) (Fig. 7C). Compared with sham rats, a greater inhibitory effect on brush-evoked responses was also notable (1-way RM ANOVA *P* = 0.0173, F_2,14_ = 5.5) (Fig. 7D). In addition, clonidine reduced levels of spontaneous firing at both doses (1-way RM ANOVA *P* = 0.0131, F_1.06,7.39_ = 10.337) (Fig. 7E), although the burst rate was only lower at the highest dose tested (1-way RM ANOVA *P* = 0.0436, F_2,14_ = 3.951) (Fig. 7F).

### 3.7. Augmenting spinal noradrenergic tone with reboxetine modestly restores inhibitory control of evoked responses in spinal nerve–ligated rats

**Figure 8.**
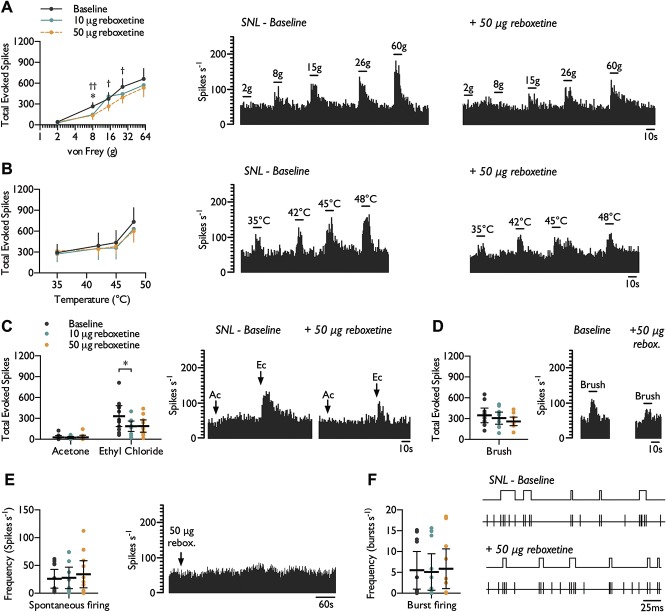
Blocking spinal noradrenaline reuptake reduces stimulus-evoked firing in the VPL of SNL rats. Wide dynamic range (WDR) neuronal responses to punctate mechanical (A), heat (B), cold (C), dynamic brush (D) stimuli, and spontaneous (E) and burst firing rate (F), before and after spinal administration of reboxetine. Histogram and event mark traces represent typical single-unit responses. Data represent mean ± 95% CI, n = 11 neurones from 7 rats. *Difference between baseline and 10-μg reboxetine, †difference between baseline and 50-μg reboxetine, *P < 0.05, **P < 0.01. Ac, acetone; CI, confidence interval; Ec, ethyl chloride; SNL, spinal nerve–ligated.

Reboxetine (10 and 50 μg), administered spinally to SNL rats, reduced mechanically evoked neuronal responses in the VPL with greater effect on lower intensity stimuli compared with noxious stimuli (2-way RM ANOVA *P* = 0.0372, F_2,20_ = 3.899) (Fig. 8A). Overall, heat-evoked responses were unaffected (2-way RM ANOVA *P* = 0.409, F_2,20_ = 0.936) (Fig. 8B), although responses evoked by noxious cooling were inhibited (acetone: 1-way RM ANOVA *P* = 0.842, F_1.2,12.01_ = 0.68; ethyl chloride: 1-way RM ANOVA *P* = 0.0346, F_1.23,12.31_ = 5.249) (Fig. 8C). In addition, there was weak evidence for a decrease in dynamic brush-evoked responses at the highest dose tested (1-way RM ANOVA *P* = 0.0276, F_2,20_ = 4.317, paired comparisons *P* > 0.05) (Fig. 8D). In marked contrast to the effects of spinal clonidine, reboxetine affected neither spontaneous activity (1-way RM ANOVA *P* = 0.187, F_1.19,11.86_ = 1.967) (Fig. 8E) nor the burst rate (Fig. 8F) (1-way RM ANOVA *P* = 0.666, F_2,20_ = 0.415).

## 4. Discussion

In this article, we describe how descending noradrenergic inhibition of spinal excitability impacts sensory coding in the ventral posterior thalamus. These data support that distinct mechanisms mediate inhibition of evoked and spontaneous neuronal activity after nerve injury. Furthermore, this distinction in inhibitory control has consequences for the actions of analgesics targeted at enhancing endogenous inhibitory tone in neuropathic conditions. To our knowledge, we also demonstrate for the first time that negating spinal noradrenergic activity through α_2_-adrenoceptors in sham rats, ie, in the absence of neuropathic injury, produces a negative affective state.

Noradrenaline in the spinal cord is derived from nuclei within the dorsolateral pontine tegmentum.^6^ NK1-positive projection neurones of the superficial dorsal horn are the origin of a spino-bulbospinal circuit, which through the parabrachial and dorsal raphe nuclei can activate descending facilitation.^58,67^ Ablation of these neurones reveals that this population also drives descending inhibition through a pontospinal loop;^55^ however, the resultant net decrease in spinal neuronal excitability indicates facilitation predominates. Significant engagement of noradrenergic inhibitory pathways is apparent during extended periods of nociceptive activity,^20,39^ acutely after an inflammatory insult or nerve injury,^7,28^ and in response to pruritogens.^18,19^ By contrast, there is a paucity of compelling evidence for the existence of tonic noradrenergic control of spinal excitability. Contradictory findings propose that either minimal/no basal activity exists within descending noradrenergic pathways,^25,29,38,41,71^ or that, these spinally directed projections tonically suppress reflex responses and spinal neuronal responses to low-intensity stimuli.^24,54,55,59^

**Figure 9.**
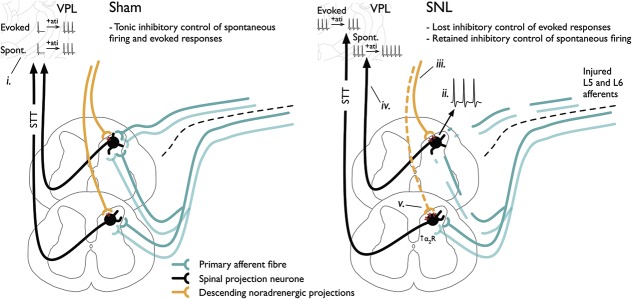
Schematic depicting proposed mechanism of noradrenergic control after peripheral nerve injury. In sham animals, the descending noradrenergic system exerts tonic inhibitory control of spontaneous and evoked firing, revealed in the VPL after spinal α_2_-adrenoceptor inhibition with atipamezole (i). After neuropathic injury, denervated spinal neurones can exhibit high levels of spontaneous firing (ii), which continue to be subjected to descending inhibitory control (iii). Disinhibition by spinally administered atipamezole results in elevated spontaneous activity in VPL (iv), whereas dorsal horn regions with conserved input have diminished descending control of spontaneous and evoked activity (v). ati, intrathecal atipamezole; spont, spontaneous firing; SNL, spinal nerve–ligated; STT, spinothalamic tract; VPL, ventral posterolateral thalamus.

We provide evidence of tonic descending inhibitory drive, which exerts inhibitory effects across sensory modalities and intensities of stimulation through α_2_-adrenoceptors, whereas in neuropathic rats, noradrenergic inhibition is partially diminished and discriminates between spontaneous and evoked activity. Previously, spinally administered atipamezole was demonstrated to increase spontaneous and evoked activity of dorsal horn lamina V/VI WDR neurones in sham rats,^54^ and corresponds with the changes in neuronal excitability observed here in the VPL. However, in SNL rats, atipamezole had no effect on either evoked or spontaneous spinal neuronal activity.^54^ It has been reported that dorsal horn neurones without receptive fields can exhibit high levels of spontaneous firing after peripheral nerve injury.^11,65^ Likewise, after amputation or in deafferentation pain, thalamic neurones without receptive fields but within the region of representation of the injury can exhibit high rates of burst and spontaneous firing.^35,36,57,72^ We hypothesise that after peripheral nerve injury, ectopic neuronal events in denervated regions of the dorsal horn are actively supressed through a postsynaptic α_2_-adrenoceptor–dependent mechanism and disinhibition by spinal atipamezole results in increased spontaneous activity in the VPL. By contrast, in regions of the dorsal horn where peripheral innervation is conserved despite nerve injury, descending noradrenergic inhibitory influences seem minimal as is evident by the lack of change in evoked neuronal responses in the VPL and dorsal horn after spinal atipamezole application (Fig. 9). Our data suggest that, akin to data on diffuse noxious inhibitory controls,^3^ the descending noradrenergic pathway is less active after neuropathy but remains intact. The conserved integrity of this pathway is further illustrated by the selective activation of spinally projecting locus coeruleus neurones because antinociceptive effects can still be produced in neuropathic states.^25^

Both animal and human data suggest that sensory and affective dimensions of pain are partially separable and implicate the anterior cingulate cortex in mediating the aversive qualities of pain.^30,53,56^ After sham surgery, conditioned avoidance behaviours were precipitated by block of spinal α_2_-adrenoceptors, which would be consistent with disinhibition within medial projection pathways converging onto the anterior cingulate cortex. The concurrent increase in spontaneous activity within the STT-VP-S_1-2_ pathway may reflect the sensory component, although it is unclear whether this represents noxious inputs. By contrast, intrathecal atipamezole failed to induce CPA in SNL rats. As neuropathic animals experience an ongoing aversive pain state, an increase in spontaneous firing within lateral but not medial pathways could underlie a failure to drive avoidance learning behaviours because sensory but not affective processing is enhanced by inhibition of spinal α_2_-adrenoceptors. The effects of atipamezole on withdrawal thresholds are comparable with our previous observations with intrathecal yohimbine.^12^ The disparity between the effect of intrathecal atipamezole on reflex withdrawal responses and evoked neuronal correlates likely reflects differences in processing mechanisms. Spinally mediated reflex withdrawals are surmised to represent sensory processing, but it is difficult to dissociate the sensory and motor components. In sham rats, the observed leftward shift in the stimulus-response relationship of WDR neurones was perhaps not sufficient to elicit enhanced motor responses. Consequently, the sensorimotor endpoints should be interpreted cautiously, and the neuronal measures may provide a more sensitive readout of subtle changes in sensory processing.

We further examined the effects of clinically used approaches to augmenting spinal noradrenergic tone: enhancing synaptic noradrenaline and directly activating α_2_-adrenoceptors. Diminished inhibitory control of evoked responses in neuropathic animals was weakly restored by spinal reboxetine in a modality-selective manner, but spontaneous activity was unaffected. The latter observation may reflect a “ceiling effect” as blocking reuptake of noradrenaline fails to enhance the high levels of basal activity within this pathway, which were revealed by atipamezole (Fig. 9). The modality-selective inhibition of mechanical and cold-evoked responses and weaker effects on heat-evoked responses implies preferential engagement of inhibitory receptors on subsets of primary afferent terminals. In the dorsal horn, these are predominantly α_2A_ receptors that colocalise with substance *P*,^62^ and α_2A_ receptors are differentially upregulated in fibre subtypes after injury.^5^ Altered receptor density could in turn affect the ability of noradrenaline to inhibit Aδ- and C-fibre–evoked transmission.^31^ It is also possible that these modest inhibitory effects are influenced by descending serotonergic facilitatory drive because, in neuropathic states, increased spinal 5-HT_3_ receptor activation can mask low levels of residual noradrenergic inhibition.^3^

Spinal clonidine has been used clinically as an adjunct or in isolation for amelioration of evoked and ongoing pain,^16^ and can be beneficial in patients with refractory cancer pain particularly those with neuropathic characteristics.^15^ In sham and SNL rats, clonidine exhibited inhibitory effects across sensory modalities and intensity of stimulus, and would be consistent with a predominantly postsynaptic inhibitory effect in the dorsal horn. Both spinally applied noradrenaline and clonidine induce postsynaptic outward currents in superficial dorsal horn neurones in vivo, in addition to decreasing the frequency of pinch-evoked excitatory postsynaptic currents.^61^ Presynaptic inhibitory effects can be mediated through α_2A_ receptors on primary afferents,^62^ α_2C_ receptors expressed by excitatory interneurones,^47^ and at higher doses, α_1_ receptors on inhibitory interneurones are potentially activated.^2^ The increased potency of α_2_-adrenoceptor agonists in neuropathic states implies plasticity within spinal circuits after an injury.^66,76^ In particular, this could relate to increases in α_2_-adrenoceptor density and enhanced receptor coupling to G-proteins to compensate for a loss of inhibitory tone due to hypoactivity in the descending pathway.^4,5,63^ Intrathecal clonidine alleviated ongoing pain and concomitantly activated reward pathways in SNL, but not sham-operated rats,^32,40,74^ and similarly, intrathecal reboxetine produced conditioned place preference in a manner dependent on pathophysiological state.^27^ Speculatively, intrathecal clonidine and reboxetine may have distinct actions on sensory and affective processing. However, a potential confound of this study is the rapid absorption of clonidine; inhibitory effects could be attributed to supraspinal mechanisms as direct actions within the locus coeruleus will impact both ascending and descending modulation of sensory transmission.^22,42,81^

In addition to proving a readout of “bottom-up” processing mechanisms, thalamic relay neurones are subjected to “top-down” processing. Corticofugal outputs can modulate sensory transmission,^43^ and thalamo-cortical neurones send collaterals to the thalamic reticular nucleus, which in turn exerts GABAergic inhibitory influences on the thalamus.^52^ Burst propensity can be regulated by tonic GABA_A_ receptor activation,^10^ and the changes in burst firing rates observed in this study may largely be determined by these circuits as a consequence of altered ascending spinal output. In addition, noradrenergic projections from the midbrain can influence neuronal excitability. In slice preparations, noradrenaline mediates a switch from burst to tonic firing patterns.^49^ This pathway provides a putative mechanism by which the state of alertness or arousal can influence sensory transmission.^14^ It is also possible that inhibition of the pontospinal pathway leads to an imbalance in the multilevel modulation by the noradrenergic system and alters ascending sensory signalling.

In conclusion, these data may have implications for the clinical approach of enhancing endogenous inhibitory activity. For those patients with small and/or large fibre neuropathy, where deafferentation of spinal neurones is likely, a significant component of ongoing pain may be driven by ectopically firing spinal neurones. Overall, patient outcomes for SNRIs could remain poor given the inability of spinal reboxetine to supress aberrant spontaneous neuronal activity in this model. Interestingly, inefficient CPM in diabetic neuropathy patients correlated with relative preservation of large afferents and advocates continued sensory profiling on this basis because there is an increased likelihood of benefit from SNRIs in this patient group.^79^ Subtype-specific α_2_-adrenoceptor agonists could be the focus of future investigations to assess whether spinal and supraspinal mechanisms can be dissociated.

## Conflict of interest statement

A.H. Dickenson has received research funding and honoraria for lectures from Grünenthal GmbH. The remaining authors have no conflict of interest to declare.

This study was funded by the Wellcome Trust Pain Consortium (102645—Defining pain circuitry in health and disease) and the National Institutes of Health (DA 041809).

## Supplementary Material

SUPPLEMENTARY MATERIAL

## Appendix A. Supplemental digital content

Supplemental digital content associated with this article can be found online at http://links.lww.com/PAIN/A596.
