# Supplementary material for: Selective deficiencies in descending inhibitory modulation in neuropathic rats: implications for enhancing noradrenergic tone
Source: Pain. 2018 Jun 29;159(9):1887–99. doi: 10.1097/j.pain.0000000000001300 (PMC6095727; doi:10.1097/j.pain.0000000000001300)
Supplement: SUPPLEMENTARY MATERIAL [file jop1-159-1887-s0001.docx]

|  | 50 μg clonidine | | 100 μg clonidine | | |
| --- | --- | --- | --- | --- | --- |
|  | Sham | SNL | Sham | SNL |  |
| Brush | -0.099 | -0.534 | -0.316 | -1.393 |  |
| 2g | 0.171 | -0.087 | -0.521 | -0.580 |  |
| 8g | -0.319 | -1.946 | -0.956 | -1.911 |  |
| 15g | -0.307 | -1.654 | -1.030 | -2.045 |  |
| 26g | -0.438 | -2.418 | -1.170 | -2.504 |  |
| 60g | -0.611 | -1.533 | -1.326 | -2.453 |  |
|  |  |  |  |  |  |
| 35°C | 0.002 | -0.081 | -0.176 | -0.442 |  |
| 42°C | 0.006 | -0.651 | -0.411 | -0.513 |  |
| 45°C | -0.201 | -1.077 | -0.443 | -0.750 |  |
| 48°C | -0.346 | -2.000 | -0.653 | -2.286 |  |
|  |  |  |  |  |  |
| Acetone | -0.302 | -0.733 | -0.761 | -0.586 |  |
| Ethyl chloride | -0.754 | -1.150 | -0.504 | -1.120 |  |
|  |  |  |  |  |  |
| Spontaneous firing | -0.863 | -0.811 | -1.318 | -1.115 |  |
| Burst rate | 0.103 | -0.343 | -0.586 | -0.931 |  |

**Supplementary table 1.** Standardised effect sizes (Cohen’s *d*) for 50 μg and 100 μg clonidine in sham and SNL rats. Positive values represent increased responses compared to baseline and negative values reflect inhibitory effects.
